# Towards SINEUP-based therapeutics: Design of an *in vitro* synthesized SINEUP RNA

**DOI:** 10.1016/j.omtn.2022.01.021

**Published:** 2022-02-02

**Authors:** Paola Valentini, Bianca Pierattini, Elsa Zacco, Damiano Mangoni, Stefano Espinoza, Natalie A. Webster, Byron Andrews, Piero Carninci, Gian Gaetano Tartaglia, Luca Pandolfini, Stefano Gustincich

**Affiliations:** 1 Central RNA Laboratory, Istituto Italiano di Tecnologia (IIT), 16152 Genova, Italy; 2 Area of Neuroscience, International School for Advanced Studies (SISSA), 34136 Trieste, Italy; 3 STORM Therapeutics, Babraham Research Campus, Moneta Building, Cambridge, CB22 3AT, UK; 4 RIKEN Center for Integrative Medical Sciences, Yokohama, Kanagawa 230-0045, Japan

**Keywords:** SINEUPs, RNA modifications, *in vitro* transcription, RNA therapeutics, DJ-1

## Abstract

SINEUPs are a novel class of natural and synthetic non-coding antisense RNA molecules able to increase the translation of a target mRNA. They present a modular organization comprising an unstructured antisense target-specific domain, which sets the specificity of each individual SINEUP, and a structured effector domain, which is responsible for the translation enhancement. In order to design a fully functional *in vitro* transcribed SINEUP for therapeutics applications, SINEUP RNAs were synthesized *in vitro* with a variety of chemical modifications and screened for their activity on endogenous target mRNA upon transfection. Three combinations of modified ribonucleotides—2′O methyl-ATP (Am), N6 methyl-ATP (m6A), and pseudo-UTP (ψ)—conferred SINEUP activity to naked RNA. The best combination tested in this study was fully modified with m6A and ψ. Aside from functionality, this combination conferred improved stability upon transfection and higher thermal stability. Common structural determinants of activity were identified by circular dichroisms, defining a core functional structure that is achieved with different combinations of modifications.

## Graphical abstract

**Figure:**
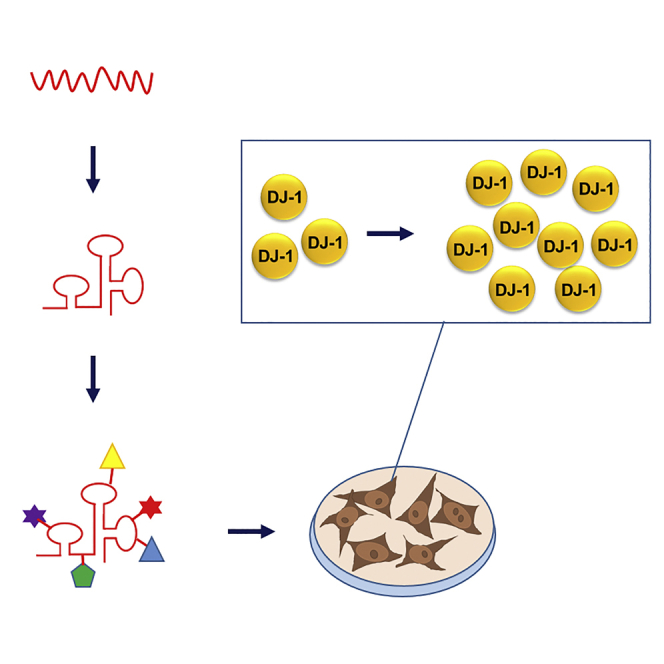

## Teaser

SINEUP RNAs are a new platform for therapeutics to increase endogenous protein expression within a physiological range. We identified a combination of modified chemical modifications to confer SINEUP activity to an *in vitro* transcribed RNA. Common structural determinants of activity were identified by circular dichroisms.

## Introduction

SINEUPs are a new class of antisense long non-coding RNAs (lncRNAs) able to enhance the translation of a target mRNA.^1^ They have a modular functional organization, in which an antisense module, called a binding domain (BD), recognizes specific target mRNAs, while a target-independent module, called an effector domain (ED), folds in a functional tertiary structure, responsible of triggering the translation enhancement.^1^, ^2^, ^3^, ^4^

The structure-activity relationship of both domains has been extensively investigated.^1^^,^^3^^,^^5^, ^6^, ^7^, ^8^, ^9^, ^10^ This information allowed the design of synthetic SINEUPs with artificial binding domains, capable of binding virtually any target gene of interest, thus defining a novel class of RNA therapeutics able to increase the protein synthesis of specific targets. Numerous works have developed synthetic SINEUPs, showing translational enhancement of a number of different target genes, both *in vitro* and *in vivo* and on ectopically expressed or endogenous transcripts, including in patient-derived cell models.^1^^,^^5^^,^^6^^,^^9^^,^^11^

Increasing understanding of the structural determinants of activity has allowed the progressive miniaturization of SINEUPs, with the aim of designing a fully functional, shorter version.^3^^,^^8^^,^^9^ Such short molecules are more convenient for drug delivery and display more predictable pharmacology and pharmacokinetics,^12^ accelerating translation to clinical applications.

Drug delivery of *in vitro* synthesized exogenous RNA faces common roadblocks linked to its susceptibility to enzymatic degradation, its potential immunogenicity, and the efficiency of uptake by the recipient cell.^13^ Such limitations are generally addressed by adding to the synthetic molecule a combination of chemical modifications (natural or artificial) in order to enhance RNA stability, minimize immunogenicity, maximize uptake, or achieve tissue-specific delivery.^14^ Various lipid formulations are often used as drug delivery carriers.^15^^,^^16^

Another important issue is the preservation of the functional secondary structure of the molecule.^17^ RNA secondary structure can be both influenced and stabilized by post- and co-transcriptional modifications.^18^ At the same time, chemical moieties exposed by the particular tertiary structure of RNA can be crucial for the functionality of the molecule, including for its interaction with RNA-binding proteins^19^ and its localization and turnover.^18^ Some modifications, such as 2′O methyl-ATP (Am),^20^ N6 methyl-ATP (m6A),^21^ and pseudo-UTP (ψ),^22^ alter substantially the RNA secondary structure, which can result in unmasking binding motifs for RNA-binding proteins.^21^

Cells add chemical modifications to newly synthesized RNA molecules during and after transcription, complementing the mere sequence of RNA with a multifaceted layer of functionalities that define the intensively studied field of epitranscriptomics.^23^ Epitranscriptomics is dynamic, with writer and eraser proteins contributing to turning on and off RNA-RNA and RNA-protein interactions by adding and removing chemical modifications.^24^^,^^25^ Although this unsteadiness complicates the spotting of modifications that are crucial for activity, it makes the study of endogenous RNA modifications even more complicated and intriguing.

Given the above considerations, when designing modular RNA drugs such as SINEUPs, it is important to define a set of chemical modifications for the general stabilization and de-immunization of the molecule but also to achieve a functional tertiary structure, which is crucial to preserve a correct network of interactions with other RNA molecules and proteins.

In order to address these issues, and to design fully functional exogenously transcribed SINEUPs, we set out to study an optimized set of modifications that could maximize the activity of an exogenous *in vitro* transcribed (IVT) SINEUP on endogenous target mRNAs.

## Results

**Figure 1:**
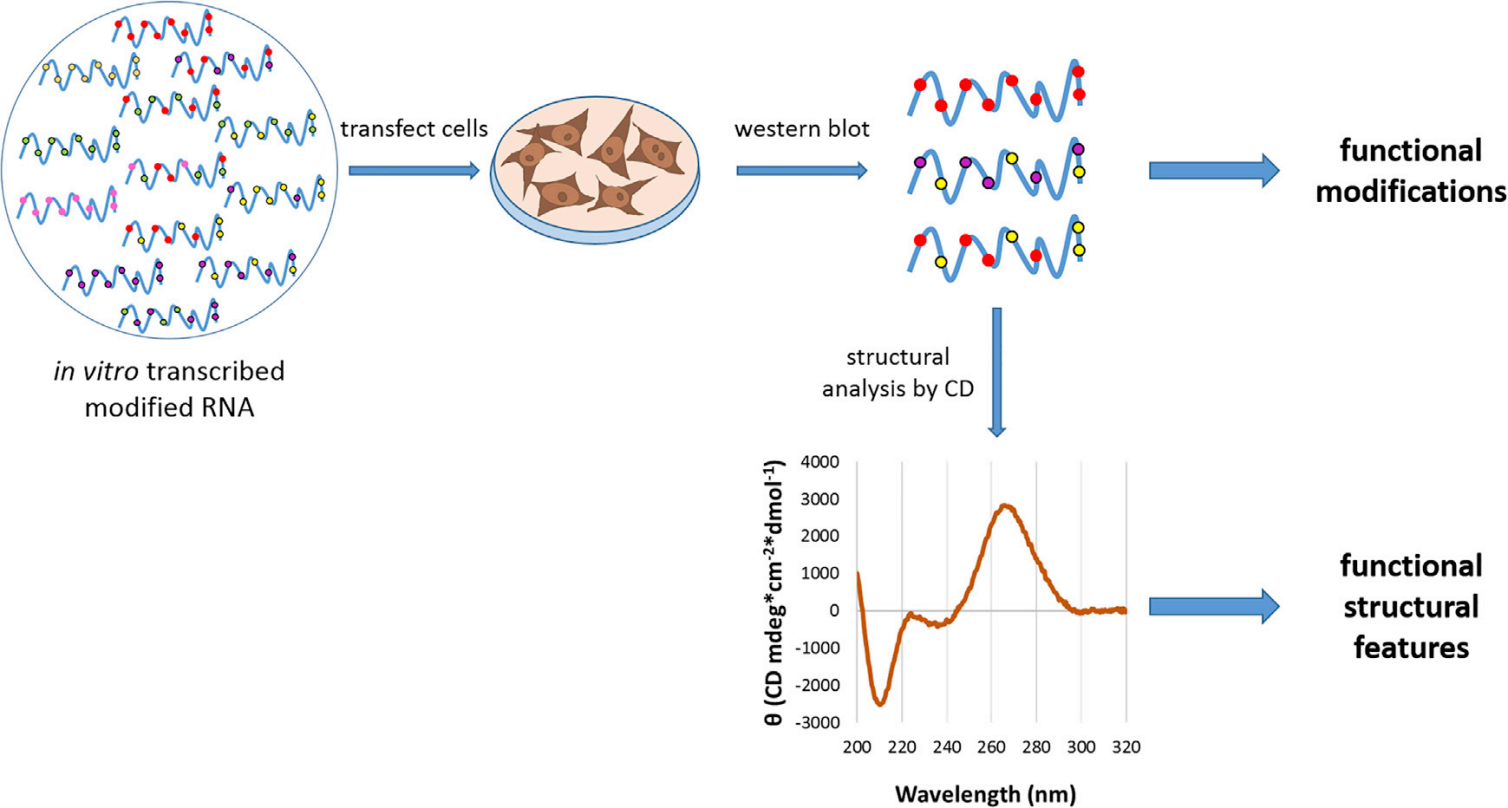
Experimental strategy IVT RNAs harboring several combinations of modifications were screened *in vitro* for SINEUP activity. This screening allowed the identification of a set of functional combinations of modifications. This selection was further analyzed using circular dichroisms (CDs) in order to identify structural determinants of activity.

IVT RNA containing different combinations of modifications were synthesized and screened *in vitro* through cell transfection and western blotting (WB) analysis of protein levels. This screening allowed the identification of a subset of exogenous modifications important for the functionality of the IVT RNA molecule. Then, structural analysis by circular dichroism (CD) unveiled structural determinants for activity. The experimental design adopted in this study is illustrated in Figure 1.

### Unmodified IVT SINEUP RNA is not functional

In order to study the functionality of modified and unmodified *in vitro* transcribed SINEUPs, we used as a model molecule a validated SINEUP, named miniSINEUP-DJ-1,^1^^,^^3^ targeting PARK7-DJ-1, a gene found mutated in familial forms of Parkinson’s disease (PD). Miniature SINEUPs (miniSINEUPs) are shorter versions of the originally discovered SINEUPs^1^ that retain the translation enhancement activity (hereafter SINEUP activity) in a more manageable molecule (∼250 nt).^3^

MiniSINEUP-DJ-1 was transcribed *in vitro* in the presence or absence of cap analog reagents, in order to obtain co-transcriptionally capped or uncapped products. Capped and uncapped transcripts were used as templates for enzymatic polyadenylation or used without further modifications. It was therefore possible to study the effect of capping and polyadenylation independently and in combination. As a control, truncated delta-BD transcripts (ΔBD), lacking the binding domain and thus not functional, were also produced.

**Figure 2:**
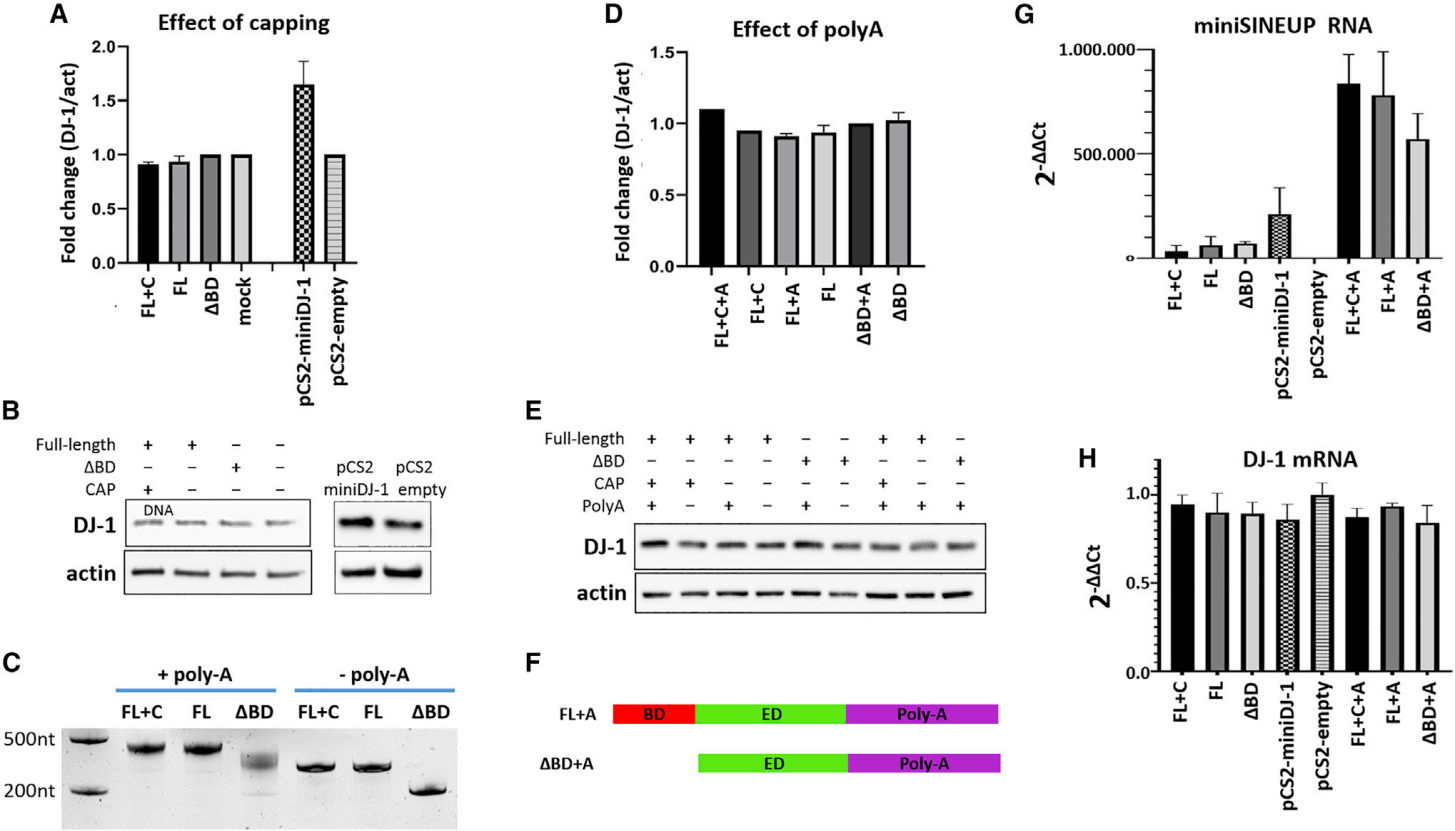
Unmodified *in vitro* transcribed SINEUPs does not increase DJ-1 protein levels (A) Average DJ-1 protein levels and (B) representative images from western blot analyses of 293T cells transfected with capped or uncapped unmodified IVT SINEUPs or with control plasmid coding for the same transcript (pCS2-miniSINEUP-DJ-1) or for no transcript (pCS2-empty). (C) PAGE image of polyadenylated SINEUPs. (D) Average DJ-1 protein levels and (E) representative images from western blot analyses of 293T cells transfected with polyadenylated and non-polyadenylated, unmodified IVT SINEUPs. (F) Scheme of IVT polyadenylated SINEUPs. (G) MiniSINEUP mRNA levels and (H) DJ-1 mRNA levels as measured using qRT-PCR. FL+C, full-length capped; FL, full-length, uncapped; ΔBD, delta-binding domain, uncapped; mock, non-transfected; FL+C+A, full-length, capped, polyadenylated; FL+A, full-length, polyadenylated; ΔBD+A, delta-binding domain, polyadenylated. Error bars: standard deviations of at least three independent experiments.

All transcripts were purified and transfected into 293T/17 cells in equimolar amounts. We first optimized RNA transfection conditions by testing different transfectants and protocols. We eventually chose to use a cationic polymer transfectant (polyethyleneimine [PEI]), as it showed the highest transfection efficiency (Figure S1). As shown in Figures 2A and 2B, both capped and uncapped miniSINEUP-DJ-1 did not cause any significant change in the endogenous levels of DJ-1 protein. Enzymatic polyadenylation added a tail of about 200 nt to capped, uncapped, and ΔBD transcripts (Figure 2C). A scheme of the final polyadenylated IVT miniSINEUP-DJ-1 and ΔBD control is shown in Figure 2F. When polyadenylated transcripts were transfected into cells, no significant changes in DJ-1 protein levels were observed for any transcript (Figures 2D and 2E), even though the level of polyadenylated RNAs was higher than the corresponding non-poly(A) counterparts (Figure 2G).

In summary, unmodified *in vitro* transcripts did not show any translation enhancements, and the addition of capping or poly(A), alone or in combination, was not sufficient to restore SINEUP activity.

### Different combinations of modifications are suitable to preserve SINEUP functionality

Given the lack of activity of unmodified IVT SINEUPs, we sought to investigate the optimal mix of modifications to preserve and maximize SINEUP activity in IVT SINEUP RNA. We screened several combinations and proportions of natural modifications, introducing modified nucleotides co-transcriptionally, by (complete or partial) substitution of the respective unmodified nucleotide with a modified analog in the reaction mixture. In particular, modifications were tested first alone and then in combination. The most promising modifications active alone were then tested in a larger number of combinations with others. A total of 22 combinations of modifications was analyzed. With this strategy, we limited progressively the magnitude of the screening, in order to focus on the most favorable combinations. This initial screening returned three “active” combinations of modifications: 2′O methyl-ATP, N6 methyl-ATP, and pseudo-UTP, which were further screened.

**Figure 3:**
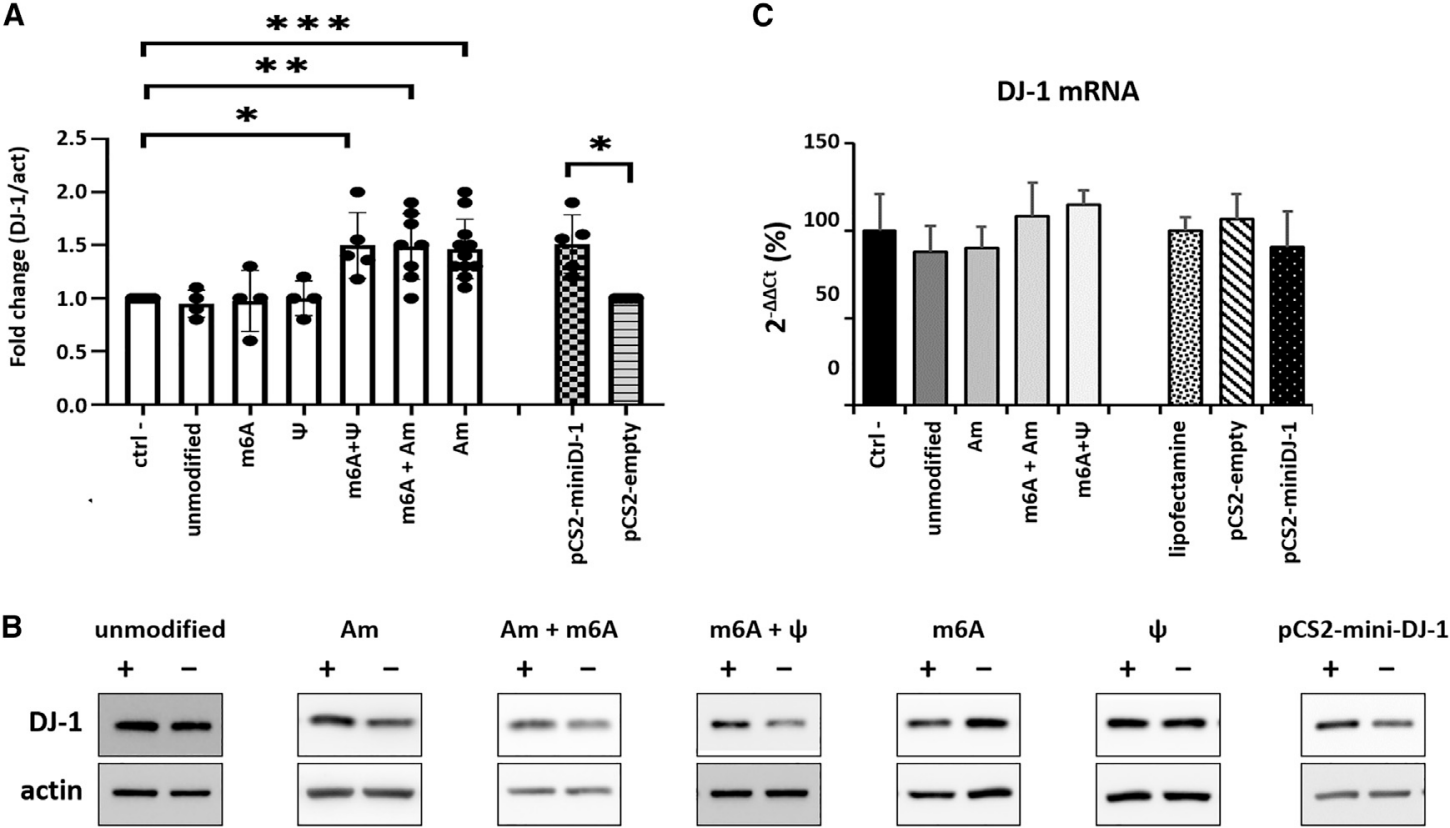
The effect of SINEUP is restored in modified IVT RNA Different combinations of modifications are suitable to preserve the functionality of miniSINEUP DJ-1. (A) DJ-1 fold change from western blot quantification of at least 3 different experiments (t test: n.s, p > 0.05; ∗p < 0.05; ∗∗p < 0.005; ∗∗∗p < 0.0005). (B) Representative western blot images of cells transfected with miniSINEUP RNA carrying different modifications or with control miniSINEUP plasmid. Negative controls are treated with the transfectant alone and no IVT RNA. (C) DJ-1 mRNA levels as measured by qRT-PCR (average of three independent experiments). Error bars: standard deviations

As shown in Figure 3, which depicts SINEUP activity as assessed using western blot, the best combinations of modifications to preserve and optimize SINEUP activity were (1) Am alone, (2) Am + m6A, and (3) m6A + ψ. As a positive control to RNA transfection, plasmid DNA coding for the same miniSINEUP was also transfected in parallel. Figure 3B includes representative WB images showing the levels of DJ-1 protein and of actin, taken as a reference gene. As shown in Figures 3A and 3B, active IVT SINEUPs increased the level of DJ-1 protein compared with control. This increase was not due to an increase in DJ-1 mRNA levels, as shown in Figure 3C, which demonstrates that the levels of DJ-1 mRNA were constant for all the samples tested.

In order to rule out any possible non-SINEUP-related effect on DJ-1 protein level, we performed an additional experiment in which we transfected cells with scrambled sequences of the same length of miniSINEUP DJ-1 and containing the same active combinations of modifications, or unmodified. As shown in Figure S2, modified scrambled sequences had no effect on DJ-1 protein levels, indicating that the SINEUP effects observed in Figure 3 are indeed due to the modified SINEUP sequence, not to any other mechanisms depending on the modifications per se. Although many modified nucleotides are incorporated by T7 RNA polymerase with an efficiency that is identical to the unmodified ones, the incorporation of certain modified nucleotides, such as 2′O-methylated ones, is inefficient^26^ (Figure S3). As a consequence, given the different kinetics of incorporation of unmodified nucleotides and nucleotides bearing different modifications,^26^ the composition of the IVT mix does not necessarily reflect the percentage of modifications found in the final molecule. For instance, when we mixed Am and m6A, we observed that the latter was incorporated by the T7 RNA polymerase several-fold more efficiently than the former. Indeed, although fully Am modified RNA was always obtained in a very low yield, demonstrating the impaired/slowed incorporation of Am in the IVT RNA, fully modified m6A RNA was always obtained in a very high yield, demonstrating the efficient/fast incorporation by the T7 polymerase (Figure S3). The mixed Am-m6A RNA was obtained in a much higher yield than the Am RNA and almost comparably to the fully m6A modified RNA. This observation suggested that the mixed molecule contained a higher percentage of m6A than Am.

In order to better characterize the content of modifications, we performed mass spectrometry (MS) analysis of these transcripts. The results show that when using a ratio of Am to m6A of 99:1 in the IVT reaction mixture, the relative abundance of the two modifications in the final molecule was only 20:80. Importantly, IVT RNAs containing lower ratios of Am to m6A were not functional, similar to transcripts containing m6A alone, indicating that a threshold level of Am is needed for activity.

Noticeably, although m6A 100% + ψ 100% IVT RNA was active, the corresponding transcripts modified with only one of these two nucleotide analogs (either m6A 100% or ψ 100%) were completely inactive (Figures 3A and 3B). In order to better understand these results, we decided to deepen our analysis by studying and comparing the stability and the structure of these modified molecules.

### RNA modifications stabilize IVT SINEUPs

The lack of activity of unmodified IVT SINEUPs could be due to reduced stability of the unmodified RNA.^27^ To address this point, we performed quantitative reverse transcriptase PCR (qRT-PCR) on total RNA extracts from cells transfected with unmodified and modified transcripts, in order to produce indications of the stability of the transfected RNAs at the endpoint of the experiment (48 h). As shown in Figure S4A, quite unexpectedly, according to these initial qRT-PCR results, unmodified IVT RNA seemed to be more stable, or to be internalized by cells much more efficiently, compared with RNA with various modifications of interest. However, it has been reported that reverse transcriptase (RT) is less efficient at producing cDNA from RNA harboring certain modifications.^28^, ^29^, ^30^ In order to avoid this potential artifact, we performed additional qRT-PCR experiments in which we added new control samples, containing total RNA extract from non-transfected cells, spiked with identical concentrations of either unmodified or modified RNAs. This allowed us to correct for any artifacts related to RT efficiency. The results in Figure S4B show that reverse transcription is severely slowed down by the presence of the modifications used in this study. RT inefficiency in polymerizing modified substrates also explains the apparent reduction in transfection efficiency of partially modified nucleotides, where transfection efficiency seems to inversely correlate to Am content in the IVT RNA (Figure S4C). In light of the above results, this effect should be interpreted as due to an impairment of reverse transcription rather than to inefficient transfection.

**Figure 4:**
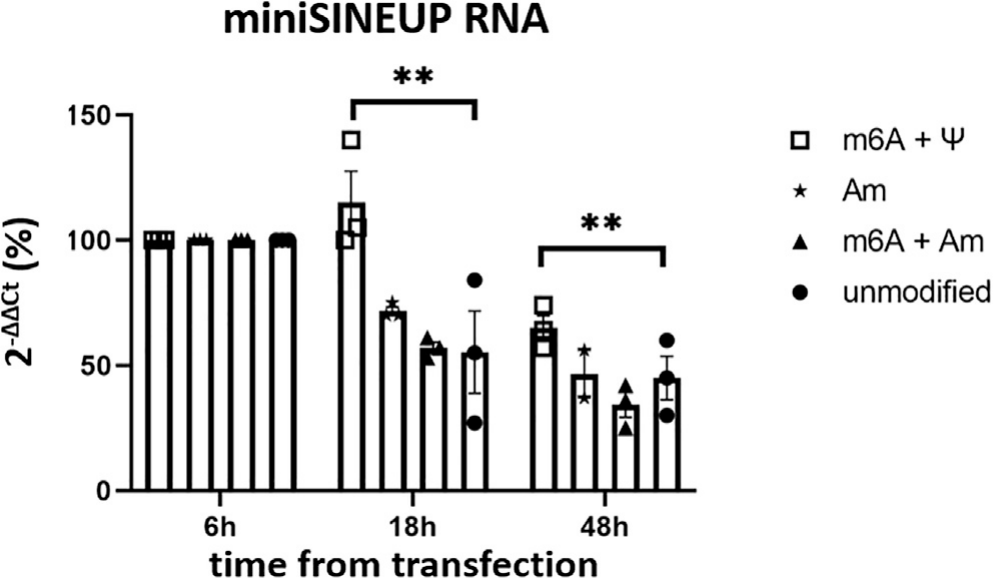
Stability of transcripts is slightly influenced by certain combinations of modifications Time course experiments demonstrate that some combinations of modifications moderately increase the stability of the IVT miniSINEUPs. SINEUP RNA levels for each modified or unmodified transcript were normalized for their respective levels at the first measured time point (6 h). Increased transcript half-life is thus only a minor determinant of SINEUP activity of modified IVT molecules (two-way ANOVA: ∗∗p < 0.005). Error bars: standard deviations of at least three independent experiments.

On the other hand, we noticed that the pattern from qRT-PCR on both RNA extracts from transfected cells (Figure S4A) and from non-transfected cells spiked with the IVT RNAs (Figure S4B) was not similar. Rather, the differences between unmodified and modified RNAs were more marked in the latter. This discrepancy may potentially be attributed to different stability of unmodified and modified RNAs at 48 h post-transfection or to a different internalization efficiency among different transcripts. In order to better clarify this point, we performed a time course experiment in which modified and unmodified IVT miniSINEUPs were transfected in equimolar amounts and cells were harvested at different time points (6, 18, and 48 h). Total RNA extracts were analyzed using qRT-PCR. In this case, as we were interested in the variation in time of each transcript rather than in their absolute amounts, any difference in the reverse transcription efficiency between modified and unmodified transcripts can be disregarded. The results in Figure 4 show that stability of unmodified transcripts dropped to less than 50% at 48 h after transfection. In contrast, in the presence of certain combinations of modifications, stability was slightly improved. This is particularly evident in the presence of the combination of m6A and ψ. However, in general the effect of modifications on stability is rather low, implying that impaired activity of unmodified IVT miniSINEUPs is caused only partially by its decreased stability due to the susceptibility to the action of intracellular nucleases.

### Secondary structure of modified IVT SINEUP and structure-activity relationship

**Figure 5:**
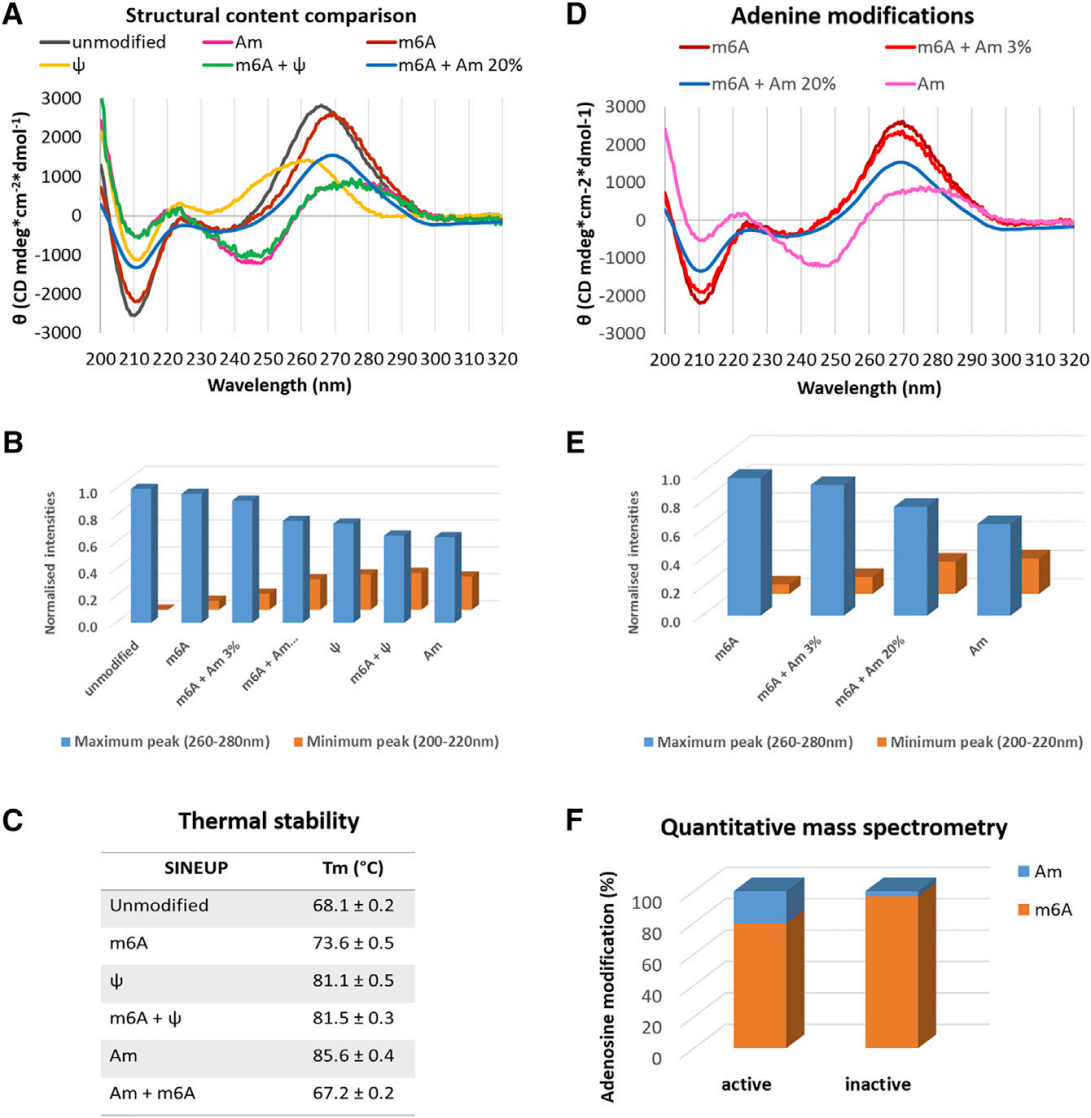
Secondary structure of modified IVT SINEUP and structure-activity relationship (A) Circular dichroism spectra of IVT miniSINEUP with various modifications highlight some structural determinants of activity: the spectra of the active IVT RNA containing Am and m6A + ψ are almost identical, identifying a core active structure; the spectra of the active m6A + Am 20% is the closest (among those analyzed) to that of the core active structure. (B) Normalized intensities of the two main peaks in the spectra in (A). (C) Thermal stability of unmodified and modified IVT miniSINEUPs. (D) Comparison of CD spectra of IVT miniSINEUPs containing different proportions of adenosine modifications. (E) Normalized intensities of the two main peaks in the spectra in (D). (F) Mass spectrometry quantification of the relative content of adenosine modifications in different IVT miniSINEUPs modified with Am + m6A mixtures and their correlation with SINEUP activity.

Circular dichroism spectroscopy was used as a tool to compare the conformations adopted in solution by the RNA molecules under investigation and to evaluate the possible role played by the structure on SINEUP activity (Figures 5A and 5B and S5). Although the complex and varied three-dimensional (3D) conformations adoptable by RNA make the identification of each secondary structure highly challenging, CD spectra in the range of 200–320 nm are extremely sensitive in offering an overall understanding of nucleic acid folding.^31^^,^^32^

The unmodified SINEUP sequence showed the typical CD profile of an A-form RNA^33^: a maximum around 265 nm indicates the presence of right-handed helices, and a minimum at 210 nm suggests a parallel orientation of the double-stranded regions. Comparable spectra were recorded for the RNA sequences comprising the m6A base alone.

When m6A was present in combination with the Am modification on the ribose, however, a decrement in the intensity of both maximum and minimum compared with the unmodified miniSINEUP was evident, hinting at a possible contribution of other conformational arrangements (Figure 5A). Noticeably, this decrement was more marked when the ratio of Am to m6A increased. The spectra of transcripts containing the “active” Am-to-m6A ratio of 20:80 showed a decrease in the 265 nm peak of 41% compared with the spectrum of transcripts containing m6A alone, while the spectra of transcripts containing the inactive ratio of 3:97 showed a decrease in the same peak of only 9.7%. Thus, the increased percentage of Am from 3% to 20%, as measured using mass spectrometry (Figure 5F), was reflected by a CD spectrum that differed more markedly from that of the IVT RNA fully modified with m6A (inactive) and resembled that of the RNA fully modified with Am (active) (Figures 5D and 5E).

Strikingly, the fully Am modified sequence and the one containing m6A in combination with ψ showed almost identical CD spectra, which markedly differed from that of the unmodified RNA (Figures 5A and 5B). Such modified molecules share similar functionality, despite bearing different modifications. Their spectra were characterized by a broader, less intense maximum in the 270–280 nm area and a minimum of comparable intensity at 245 nm, indicating spectral features similar to that of B-form DNA,^34^ rather than the A-form, typical of RNA.^35^^,^^36^

Noticeably, the spectrum of the IVT RNA containing m6A and ψ (functional) was very different from both the one containing m6A alone and that containing ψ alone (both not functional). Indeed, the spectrum of the miniSINEUP containing m6A was very similar to that of the unmodified RNA. On the contrary, the spectrum of the RNA fully modified with ψ reflected the distortion of the helix attributable to this particular nucleotide,^37^^,^^38^ with pronounced negative band at 210 nm, a zero at around 235 nm, and a weak maximum that covers the region spanning between 240 and 237 nm. This spectrum cannot be associated to a singular conformation but rather is the overlap of several signals derived by the ψ-associated alterations on the most common conformations.

Studies of the thermal stability of the unmodified and the modified miniSINEUPs were also performed by means of CD, as structural stability is a crucial parameter when designing RNA for potential therapeutic applications (Figure 5C). The apparent melting temperature (*T*_m_) was determined by following the variation of the CD intensity at 270 nm as a function of temperature.^39^ In accordance with literature, we found that the two miniSINEUPs displaying a spectrum similar to B-form conformation of DNA showed improved stability compared with the unmodified RNA sequence and increased the *T*_m_ by about 15°C.^40^ Of the remaining SINEUP versions, Am + m6A RNA sequences had an apparent *T*_m_ comparable with the unmodified one, while the RNA with m6A displayed a *T*_m_ increment of 5°C. Apparent melting temperature curves for all transcripts are reported in Figure S6.

## Discussion

RNA post-transcriptional modifications are widespread across the transcriptome, in both coding and non-coding regions.^23^^,^^41^ To date, more than 163 post-transcriptional modifications have been identified,^23^ building on the versatility that characterizes RNA per se (arising from the secondary and tertiary structure of RNA, its short life, and its amplification through transcription), offering enormous potential for functional diversity. The development of novel methodologies, allowing transcriptome-wide mapping of some RNA modifications with single-nucleotide resolution,^42^, ^43^, ^44^, ^45^, ^46^, ^47^, ^48^, ^49^ has recently boosted revitalized attention in the scientific community on the field, which has been defined as epitranscriptomics. RNA modification landscape is cell type specific and dynamic,^50^^,^^51^ with at least 340 RNA-modifying enzymes or cofactors identified so far,^23^ including specific writer,^52^ eraser,^52^^,^^53^ and reader^52^^,^^54^^,^^55^ proteins. Such enormous modification potential allows rapid and finely tuned adaptive responses to environmental changes.

IVT RNA lacks the natural modifications present in RNA, as well as 3′ and 5′ end processing such as capping and polyadenylation, and it may thus result in both instability and inefficiency *in vivo*. To overcome these drawbacks, capping can be introduced co-transcriptionally (by partial substitution of the guanosine in the nucleotide mixture with a cap analog) or post-transcriptionally (by a step of enzymatic capping with vaccinia virus capping enzyme).^56^ Polyadenylated transcripts can be obtained by transcribing poly-thymidylated DNA templates or by enzymatic polyadenylation of RNA transcripts with a poly(A) polymerase. Here we used co-transcriptional capping and enzymatic polyadenylation on IVT miniSINEUP RNA without further nucleotide modifications and showed that the modification of RNA extremities was not sufficient, per se, to restore SINEUP activity of unmodified RNA. These results, also confirmed in a parallel study published during the preparation of this work,^57^ demonstrated the necessity to introduce modifications in the IVT miniSINEUP RNA.

Several mixes of artificial and natural modifications have been used to address stability and immunogenicity roadblocks of exogenous IVT RNA, which still represent major hurdles for the design and delivery of RNA therapeutics.^58^, ^59^, ^60^, ^61^ In this study, after a larger initial screening, we focused on a restricted set of natural modifications, which also display interesting features for an artificial exogenous RNA aimed at increasing the translation of an endogenous target mRNA, such as SINEUPs.

In particular, 2′O methyl-ATP is often found in the extended cap structure in mRNA, where the 2′O sites of the first and second nucleotides adjacent to the cap are methylated.^62^^,^^63^ The first nucleotide can be also reversibly di-methylated, bearing an additional N6 methylation (m6Am),^64^ which is important in the control of mRNA stability.^64^ Am is also important for the stability of some ncRNA.^65^ Such stabilizing effects render this modification very interesting for the synthesis of exogenous RNAs. Methylation at cap-adjacent sites also affects translation efficiency^66^ and self-non-self recognition, being thus important for the induction of self-tolerance^67^, ^68^, ^69^ or de-immunization of exogenous RNA.

Both N6 methyl-ATP and ψ are involved in post-transcriptional gene regulation.^44^^,^^50^^,^^52^^,^^70^ In particular, m6A is the most abundant internal modification in eukaryotic mRNA.^54^ It interacts with protein-binding partners to regulate transcript stability^55^ and promote translation through different mechanisms. First, it favors cap-dependent translation through interaction with YTHDF1 reader protein.^71^ Second, m6A supports an alternative cap-independent translation path, which is not IRES dependent.^72^^,^^73^ Third, di-methylated adenosine (m6Am) in the position adjacent to the m^7^G cap enhances transcript stability and promotes translation.^64^ Such important roles in translation make it an interesting candidate for supporting or regulating (possibly synergistically) SINEUP activity.

Ψ is the most abundant modification in non-coding RNA (ncRNA).^44^ It is important for the stabilization of the secondary structure,^37^ which makes it interesting for the design of a ncRNA that functions through structural domains, such as SINEUPs. Moreover, ψ has been widely used in the development of RNA drugs because of its ability to reduce the immunogenicity of exogenous RNA.^27^^,^^60^

Our results show that the three modifications above are all capable of preserving SINEUP activity of IVT RNA, when present in the appropriate combination and ratio. Somehow unexpectedly, when used in combination (Figure S7), the effect of the three modifications together was not additive or synergistic. This result may indeed produce different interpretations. First, the upregulation of the protein level of an endogenous protein can be controlled by feedback mechanisms that maintain its level within a physiological range, so that we cannot observe excessive over-expression of endogenous protein, as has already been reported with other SINEUPs acting on endogenous targets *in vitro* and *in vivo*.^3^ Second, different combinations of modifications lead to the stabilization of the same core structural domain of the SINEUP, as evidenced by CD results. This core functional structure may be disrupted if an excessive number of modifications is introduced, because of a detrimental structural distortion. Third, the effect of the modifications studied on increasing nuclease resistance of the exogenous SINEUP may be similar, so that adding three modifications on the same molecules might not extend the RNA half-life more than a single modification. Interestingly, the percentage of modified nucleotides needed for activity of an IVT SINEUP may be well below 100%, as 20% of Am in the Am + m6A mixture is sufficient to restore SINEUP activity that is lacking in the 100% m6A-modified transcript (Figure 5D). This resembles the physiological situation of endogenous transcripts and is in accordance to studies on other IVT RNAs,^26^ in which a comparably small proportion of modified nucleotides is sufficient for activity.

Figure 4 shows how extended half-life of the modified IVT RNA, compared with its unmodified counterpart, explains only in part the functionality of the former. However, nuclease resistance of modified RNA is likely not the sole determinant of the activity of modified molecules. As resulted from CD analyses, structural stabilization by RNA modification^18^ is an important factor for the functionality of exogenous SINEUPs. Indeed, two of the three combinations of modifications leading to a functional SINEUP (Am and m6A + ψ, respectively) also displayed a strong structural stabilization, as evidenced by their very high apparent melting temperature. On the other hand, the third combination (Am + m6A) did not show higher thermal stability.

Interestingly, although neither ψ nor m6A modifications alone could restore the functionality of IVT miniSINEUPs, they did so when present in combination on the same molecule. The gain of activity was reflected in a marked structural change in the molecule, as evidenced by CD spectra of the mono- and double-modified transcripts. A previous work^26^ showed that the activity of fully m6A modified and ψ modified transcripts, either alone or mixed together in a sample, was markedly different from that of transcripts in which m6A and ψ were present on the same RNA molecule. Noticeably, in fully m6A + ψ double-modified transcripts, m6A and ψ are found opposite one another in each double-stranded RNA (dsRNA) portion of the molecule (as A and U pairs are substituted by m6A and ψ). As miniSINEUPs are characterized by an extensive stem-loop folding,^7^ it is tempting to hypothesize that the presence of these two modifications together has a strong influence on the active structure of the IVT miniSINEUP.

In an attempt to correlate the structural properties brought to the miniSINEUP by a given nucleotide modification and the observed SINEUP activity, we speculated that the B-like form may be the most biologically active miniSINEUP. This consideration would explain why the SINEUP versions containing Am alone, or the combination of m6A and ψ, displaying almost identical B-like conformations from CD spectra, are biologically active despite the different modification patterns. Nevertheless, this would not fully justify the activity of the miniSINEUP modified with both Am and m6A.

These data suggest that other factors contribute to SINEUP activity of the modified IVT RNAs tested in this study. For instance, RNA modifications may modulate the interaction with RNA binding partners important for activity^21^^,^^74^ or alter subcellular localization of the exogenous RNA.^74^ Currently, it remains unclear in which subcellular compartments the IVT SINEUP BD RNA binds its target, although previous results on plasmid-expressing SINEUPs showed the interaction occurred in the nucleus.^75^ Subcellular localization of modified SINEUP may thus be more favorable for SINEUP-target interaction compared with unmodified SINEUPs, leading to increased activity.

Another important aspect to take into consideration is the fact that RNA changes its conformation when it is hybridized to another RNA or DNA molecule. Given that our miniSINEUP is a modular molecule, including an antisense RNA BD and a more structured ED, the structure of the isolated domains is definitely worthy of further investigation. For instance, it has been shown that DNA-RNA hetero-hybrids have a conformation that is intermediate between A-form RNA and B-form DNA, while when such hetero-hybrids include fully 2′O-modified RNA, the resulting conformation corresponds fully to A-form RNA.^76^ Adjacent modifications could lead to more homogeneous structures because of a lower frequency of structural transitions.^76^ It is likely that the contribution of modifications in each of the two domains is different and that the two SINEUP domains require specific sets of modifications in order to stabilize, for instance, the hybridization with RNA in the BD or to stabilize a certain secondary folding in the ED. For instance, it has been shown that methylated bases can strongly favor the formation of hairpin structures,^77^ a feature of the SINEUP ED.^7^ All these aspects should be taken into account when designing modular RNA therapeutics.

In summary, the results obtained using CD spectroscopy support the theory that a common secondary structure may be linked to comparable SINEUP activity. In addition, certain modifications (such as m6A) may also contribute to SINEUP activity by contributing to regulate the interactions with protein or RNA binding partners.

The modularity of SINEUP molecules also aids in generalizing our results obtained on the model miniSINEUP DJ-1. Indeed, as already mentioned, the ED is universal for all the SINEUPs, whereas the BD is different and target dependent. As the BD is the unstructured module of the molecule, because of the hybridization with the target RNA, it is reasonable to infer that the functional secondary structure of the ED is a general feature of all SINEUPs, independent of their binding domain.

### Conclusions

In this study, we have defined different combinations of natural modifications that can be added to an IVT SINEUP RNA in order to reproduce the functionality of an endogenously transcribed SINEUP. Among the tested modifications, the combination of m6A and ψ was the best combination. Indeed, aside from retaining SINEUP functionality, it enhanced the half-life after transfection and conferred enhanced structural stabilization. Furthermore, by structural analysis through circular dichroism, we have been able to correlate structural features with SINEUP activity, identifying a common functional structure obtained by different sets of modifications. These results further support the notion of a modular arrangement of this class of ncRNAs, in which an unstructured domain (BD), operating by sequence-specific hybridization with a target, is paired to a structured domain (ED), with specific and functional tertiary folding and modifications, operating via the interactions with protein and RNA binding partner to achieve translation enhancement. Both the correct set of chemical modifications and a functional structural arrangement are therefore important in the design of fully functional, tertiary-folded RNA therapeutics, such as SINEUPs.

## Materials and methods

### Plasmids

Plasmids for *in vitro* transcription were all based on a pCMV6 backbone. MiniSINEUP-DJ-1^3^ (Figure S8) was excised from a pCS2 scaffold using XhoI and SnaBI and cloned downstream from the T7 promoter in pCMV6 by using SalI and PmeI restriction sites to obtain pCMV6-miniSINEUP-DJ-1. The latter was used to obtain pCMV6-DeltaBD by excising the binding domain using EcoRI and re-ligating the backbone plasmid. A miniSINEUP-DJ-1 cloned into pCS2 (pCS2-miniSINEUP-DJ-1) and the corresponding pCS2-empty vector were used as control DNA in RNA transfections experiments.

### *In vitro* transcription

Unmodified and modified RNA molecules were transcribed *in vitro* using the MEGAscript T7 kit (Thermo Fisher Scientific). Modified nucleotides triphosphates (2′O methyl-ATP, N6 methyl-ATP, pseudouridine triphosphate) were purchased from TriLink Biotechnologies. *In vitro* transcription reactions were assembled according to recommendation from the kit manufacturer and incubated overnight (16 h) at 37°C. Co-transcriptional capping was performed by substituting part of the GTP in the reaction with cap analog m7G(5′)ppp(5′)G (Thermo Fisher Scientific). After transcription, capped transcripts were treated with 1 μL thermosensitive shrimp alkaline phosphatase (tSAP; Promega) for 15 min at 37°C. All capped and uncapped transcripts were then treated with DNase I for 15 min at 37°C and immediately purified using the RNeasy Mini Kit (Qiagen). Post-transcriptional polyadenylation was performed on purified transcripts by adding 1 μL *E. coli* poly(A) polymerase (5 U/μL; New England Biolabs) and 1 mM ATP and incubating at 37°C for 30 min. Polyadenylation reactions were stopped by purification with the RNeasy Mini Kit. All transcripts were checked for purity and integrity by UV-vis spectrophotometry and denaturing poly-acrylamide gel electrophoresis (PAGE).

### Cell lines and transfections

293T/17 cells, purchased from the American Type Culture Collection (ATCC), were cultured in DMEM high glucose (4.5 g/L D-glucose) with L-glutamine (Gibco), completed with 10% fetal bovine serum and 1% penicillin/streptomycin mix and 1% HEPES buffer (Gibco). 293T/17 cells were passaged 1:5 to 1:10. Cell lines were used within passage 10.

RNA was transfected using polyethyleneimine (MW 25000, branched) (catalog #408727; Sigma) according to the following protocol. Cells were plated at a density of 250,000 per well in a six-well plate 24 h before transfection, in DMEM complete medium. The day after, immediately before transfection, medium was replaced with 1 mL Opti-MEM (Thermo Fisher Scientific) per well. A transfection mix was prepared containing 400 ng RNA in 160 μL DMEM without serum and antibiotics, at room temperature. PEI (2.5 μL, 40 μM) was added to the reaction, and the tube was briefly vortexed for 1 s and incubated at room temperature for 10 min. The tube was vortexed again for 1 s and added to the cells. Cells were harvested at 48 h for western blot and qRT-PCR analyses.

DNA control transfections were carried out in parallel using 1 μg plasmid DNA (pCS2-miniSINEUP-DJ-1 or pCS2 empty) and 3 μL Lipofectamine 2000 (Thermo Fisher Scientific) in 200 μL Opti-MEM. In this case, transfection medium was changed 6 h after transfection and replaced with 2 mL DMEM complete medium per well. Cells were harvested at 48 h.

### Western blotting

Cell pellets were lysed in lysis buffer (PBS + 1% Triton X100) with cOmplete protease inhibitor (Roche) on ice, briefly sonicated on ice, and centrifuged at maximum speed for 20 min at 4°C. Supernatants containing total lysates were collected on ice and quantified for total protein contents using the BCA assay kit (Thermo Fisher Scientific). Total lysate (10 μg) was loaded on NuPAGE 10% Bis-Tris, 1.5 mm, Protein Gel, 10-well (Thermo Fisher Scientific) and run at 120 V for approximately 90 min for SDS-PAGE. Gels were transferred to a 0.2 μm nitrocellulose membrane (Amersham) at 250 mA for 90 min. DJ-1 was detected with mouse anti-DJ-1 primary antibody (Enzo Life Sciences), and actin was detected with rabbit anti-β-actin primary antibody (Sigma Aldrich), both diluted 1:8,000 in 5% BSA in Tris-buffered saline-Tween 20 (TBST) and incubated overnight at 4°C. Horseradish peroxidase conjugated secondary anti-mouse and anti-rabbit antibodies were diluted 1:10,000 and incubated at room temperature for 1 h. Signals were detected using Pierce ECL plus detection reagent (Thermo Fisher Scientific) and read on ChemiDoc (Bio-Rad). Band intensities were calculated using ImageJ (NIH) and Image Lab (Bio-Rad) software. Data from a minimum of three independent experiments were analyed statistically using t tests.

### Quantitative reverse transcriptase PCR

Total RNA was extracted using the RNeasy Mini Kit, and samples were then quantified using UV-vis spectrophotometry. DNase digestion was then performed adding 1 μL Turbo DNase I (Sigma Aldrich) and 1 μL 10× DNase I buffer to 800 ng RNA in a 10 μL reaction and incubating for 15 min at room temperature. DNase was inactivated by adding 1 μL DNase I stop solution and incubating for 10 min at 70°C. DNase-treated RNA (5 μL, approximately 400 ng) was reverse transcribed using iScript cDNA synthesis kit (Bio-Rad) according to the manufacturer’s instructions, in a 20 μL reaction. qRT-PCRs were then set up, including 2 μL cDNA, 5 μL iTaq Universal SYBR Green Supermix (Bio-Rad), 0.4 μL forward primer, and 0.4 μL reverse primer, in a total volume of 10 μL. qRT-PCR was run on a CFX96 Touch Real-Time PCR Detection System (Bio-Rad) using the following thermal cycling conditions: 98°C for 30 s, then 40 cycles at 95°C for 10 s and 60°C for 30 s. Glyceraldehyde 3-phosphate dehydrogenase (GAPDH) gene was used as an internal reference to normalize the results. Data from a minimum of three independent experiments were analyzed statistically using two-way ANOVA or t tests as appropriate.

### Mass spectrometry

Isolated RNA was digested to component nucleosides with an enzyme cocktail of Benzonase, phosphodiesterase, and alkaline phosphatase (Merck), all used according to the manufacturer’s instructions. Nucleosides were separated by reverse-phase liquid chromatography; eluent A was 0.1% v/v formic acid in water, and eluent B was 0.1% v/v formic acid in acetonitrile, and a non-linear gradient of 2%–15% B resolved nucleosides across an Acquity HSS T3 C18 column (Waters). The eluent was sprayed into a 4500 triple quadrupole mass spectrometer (Sciex) and characterized using tandem mass spectrometry with a multiple reaction monitoring approach. Injection amounts were normalized by internal calibration with isotopically labelled uridine and quantification extrapolated from external calibration of a range of nucleoside standards using MultiQuant software (Sciex).

### Circular dichroism

Far- and middle-UV CD spectra of RNA molecules were recorded on a JASCO-1500 spectropolarimeter using a 1 mm path length quartz cuvette and a constant N_2_ flow of 4.0 L/min. RNA was defrosted immediately before the experiments and diluted in 10 mM phosphate buffer at pH 7.2 with 5 mM MgCl_2_ to reach a concentration between 100 and 150 ng/μL. CD spectra were acquired in duplicate or triplicate at 25°C and are reported as the average of at least 10 scans. All spectra were blanked for the buffer signal, corrected for the concentration and number of nucleotides, and expressed as molar ellipticity. The thermal denaturation analysis was performed monitoring the changes of the CD signal at 270 nm between 25°C and 100°C, using a 1°C/min gradient. The apparent melting temperature was derived by fitting the data according to the literature.^78^

## Supplemental information

Document S1. Figures S1–S8

Document S2. Article plus supplemental information
