# Supplementary material for: Towards SINEUP-based therapeutics: Design of an in vitro synthesized SINEUP RNA
Source: Mol Ther Nucleic Acids. 2022 Feb 2;27:1092–102. doi: 10.1016/j.omtn.2022.01.021 (PMC8857549; doi:10.1016/j.omtn.2022.01.021)
Supplement: Document S1. Figures S1–S8 [file mmc1.pdf]

OMTN, Volume *27*

Supplemental information

Towards SINEUP-based therapeutics: Design of an *in vitro* synthesized SINEUP RNA

Paola Valentini, Bianca Pierattini, Elsa Zacco, Damiano Mangoni, Stefano Espinoza, Natalie A. Webster, Byron Andrews, Piero Carninci, Gian Gaetano Tartaglia, Luca Pandolfini, and Stefano Gustincich

SUPPORTING INFORMATION

Towards SINEUP-based therapeutics: design of an *in vitro* synthesized SINEUP RNA

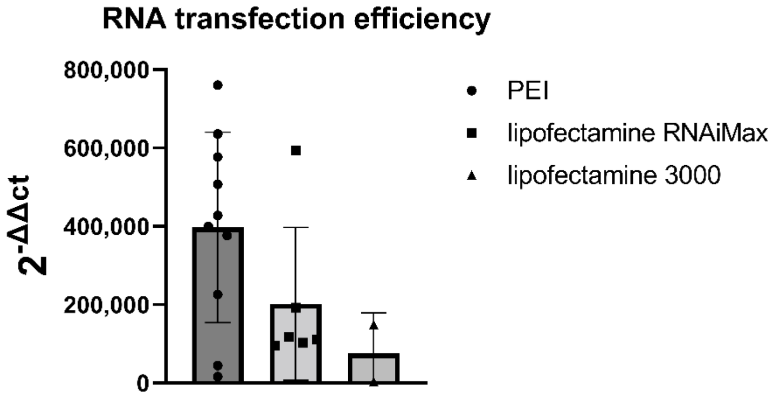

Figure S1: Optimization of transfection efficiency. Unmodified miniSINEUP RNA was transfected with different transfectants in independent experiments. Cells were harvested at 48h and transfection efficiency was measured by RT-qPCR and calculated with the ΔΔCt method. PEI was the most efficient transfectant.

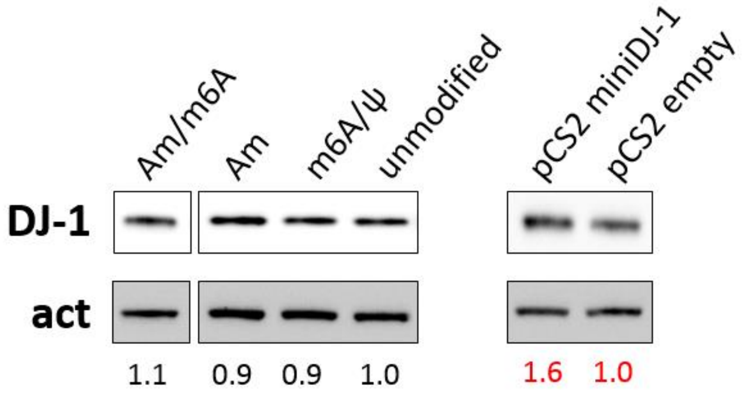

Figure S2: Both unmodified and modified scrambled SINEUPs have no activity. Representative western blot images of cells transfected with a scrambled SINEUP RNA carrying different modifications, or with miniSINEUP plasmids as a positive control. Numbers below the images indicate the average fold change (calculated from 3 different experiments) in the level of DJ-1 as compared to negative controls (unmodified scrambled RNA or pCS2 empty plasmid).

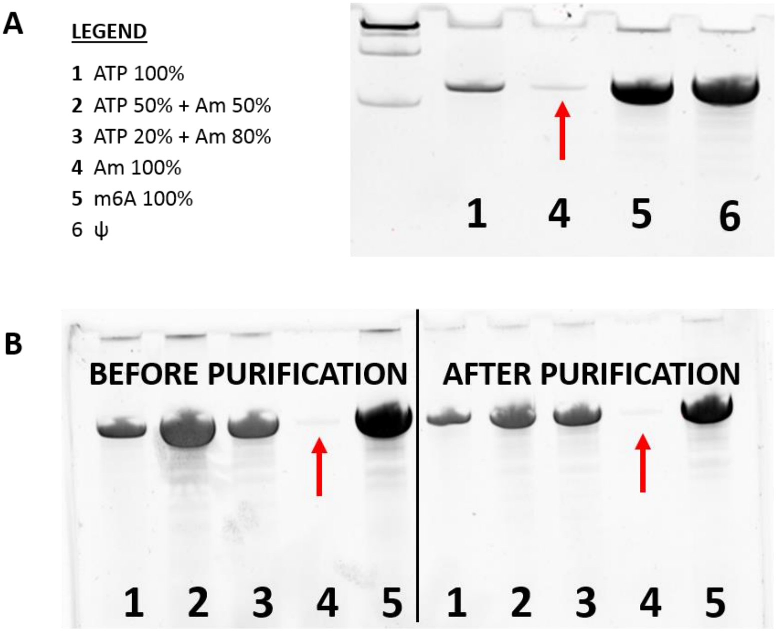

Figure S3: Optimization of transcription of modified RNAs. A) Incorporation of Am with T7 RNA polymerase is inefficient, as evidenced by denaturing PAGE. **B)** Low yield of Am-modified RNA does not depend on modified transcript loss during RNA purification (red arrows).

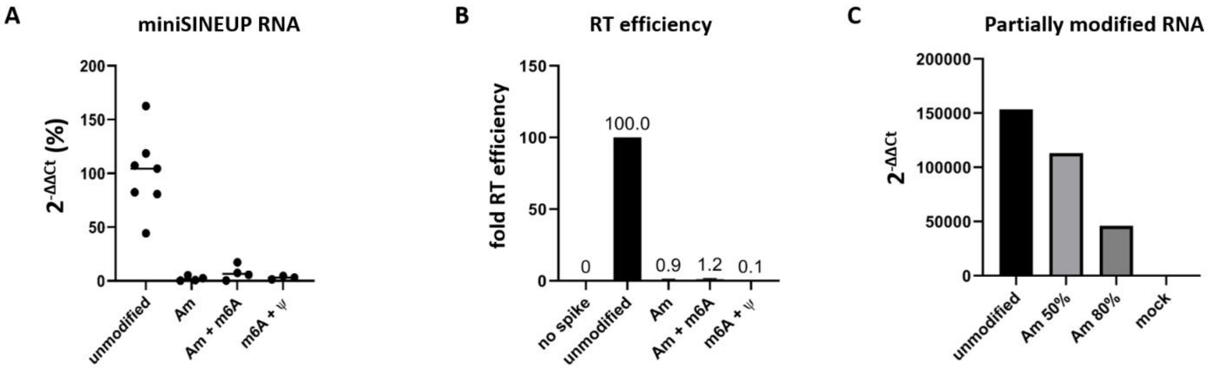

Figure S4: inefficient reverse transcription of modified nucleotides. A) normalized gene expression data from RT-qPCR on total RNA extract of 293T/17 cells transfected with unmodified and modified IVT SINEUPs. Total RNA is extracted at 48h after transfection. Input data for normalization: average of gene expression data from samples transfected with unmodified IVT RNA. B) RTqPCR on total RNA extracts from non-transfected cells spiked with equal amounts of modified and unmodified IVT RNA shows that reverse transcription is severely impaired in the presence of certain modifications. C) Reverse transcription efficiency inversely correlates with the percentage of modified nucleotides. Transcription efficiency, as measured by RT-qPCR with the ΔΔCt method, apparently decreases with increasing percentage of modified NTPs in the transcript. The effect is instead an artefact caused by inefficient reverse transcription of modified substrates

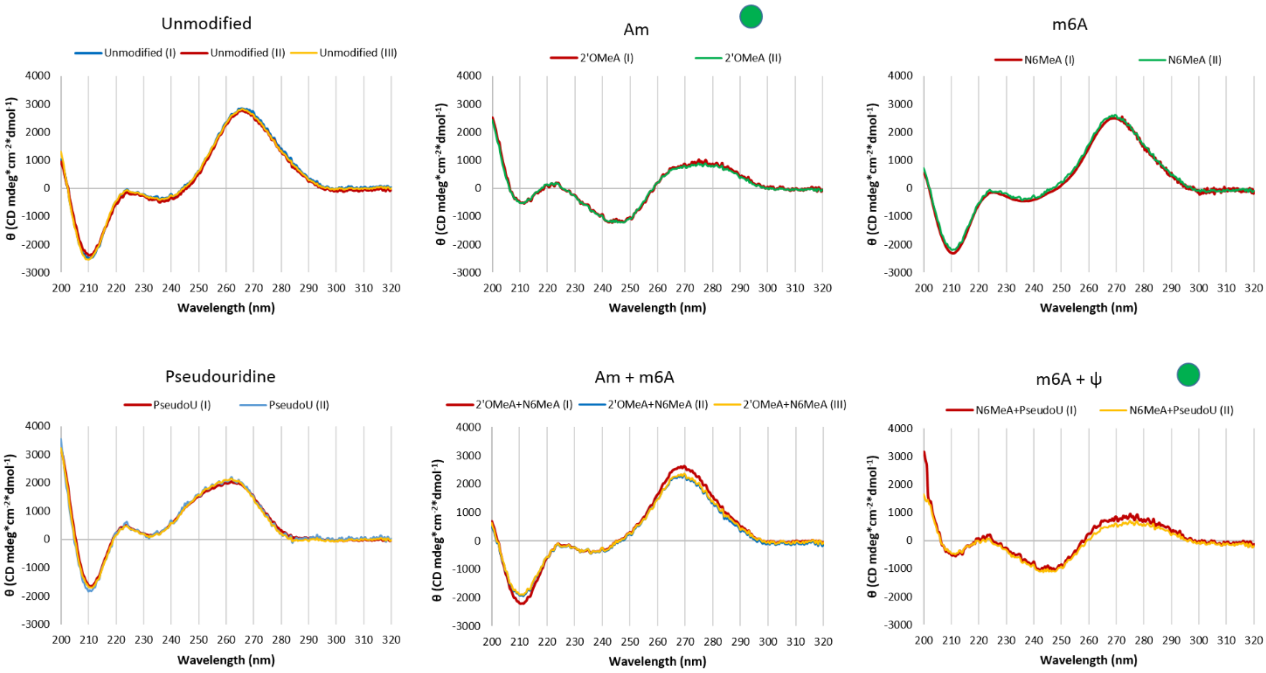

Figure S5: Circular dichroism spectra of unmodified and modified miniSINEUPs. All spectra have been acquired two times on independent syntheses of IVT miniSINEUPs and showed reproducible signals. The green dots mark functional miniSINEUPs

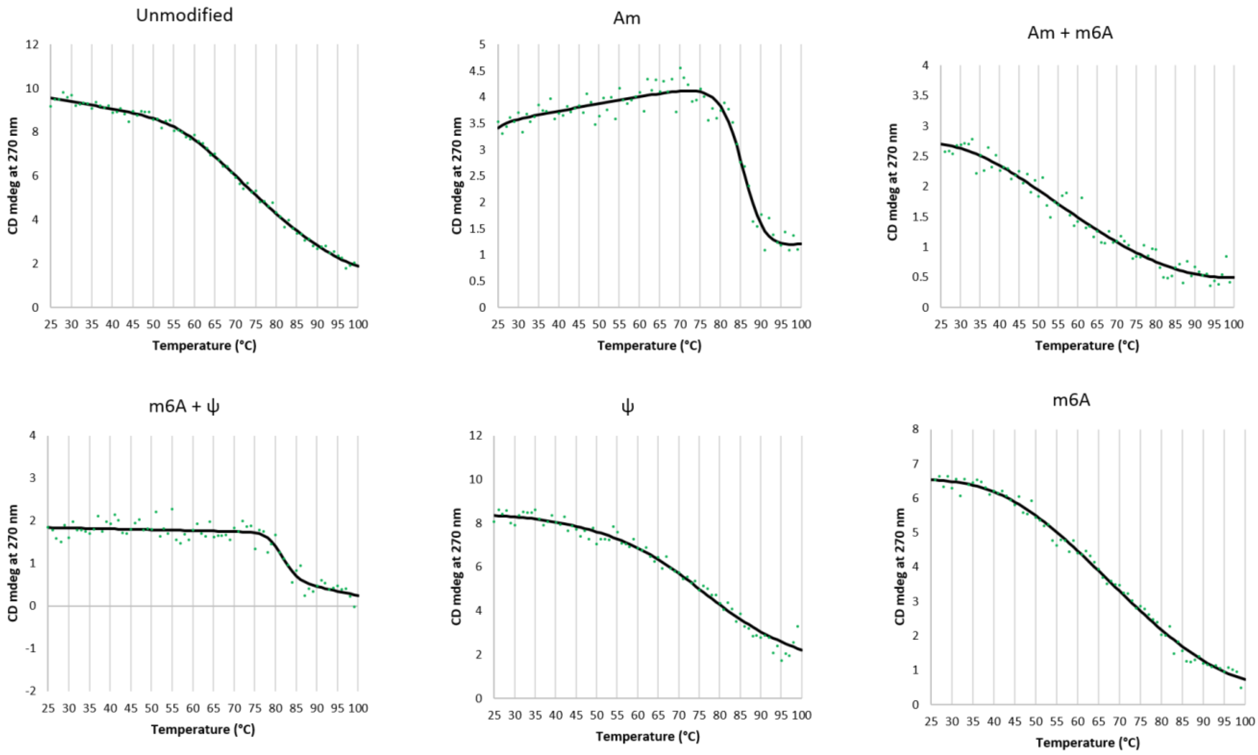

Figure S6: Apparent melting temperature curves of unmodified and modified SINEUPs as obtained by circular dichroisms.

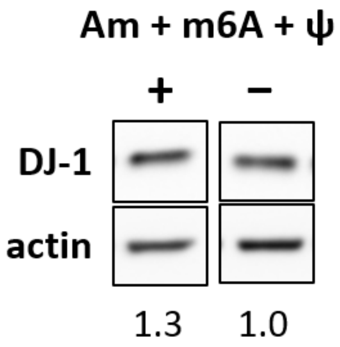

Figure S7: The combination of the three "active modifications in the same IVT RNA molecule does not further increase SINEUP activity. Representative western blot images of DJ-1 and actin proteins levels in total protein lysate from 2937/17 cells transfected with IVT RNA modified with Am, m6A and ψ, as compared to control cells.

A) Sequence of miniSINEUP DJ-1

```
5′TCGAGCCATTTTTATGTTAT
ATGTTTACAAGCCCCACACCAG
GCTGAAAATCTGCAGAATTCGC
CCTTCAGTGCTAGAGGAGGTCA
GAAGAGGGCATTGGATCCCCCA
GAACTGGAGTTATACGGTAACC
TCGTGGTGGTTGTGAACCACCA
TGTGGATGGATATTGAGTTCCA
AACACTGGTCCTGTGCAAGAGC
ATCCAGTGCTCTTAAGTGCTGA
GCCATCTCTTTAGCTCCAAGCT
T3′
```

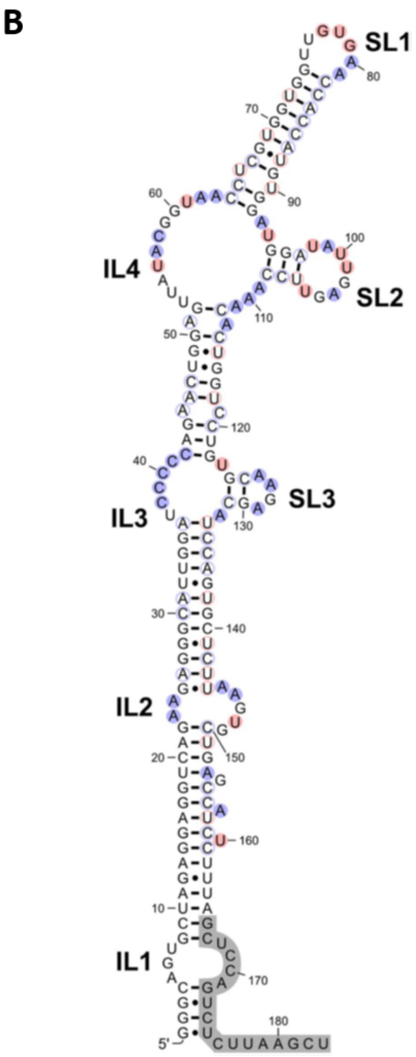

Figure S8: miniSINEUP DJ-1: A) sequence and B) predicted tertiary structure (reproduced without any changes from Podbevsˇ ek *et al*., Scientific Reports 2018[1], link to the Creative Commons license: https://creativecommons.org/licenses/by/4.0/).

REFERENCES

1 Podbevsek, P., Fasolo, F., Bon, C., Cimatti, L., Reisser, S., Carninci, P., Bussi, G., Zucchelli, S., Plavec, J., and Gustincich, S. (2018). Structural determinants of the SINE B2 element embedded in the long non-coding RNA activator of translation AS Uchl1. Scientific reports *8*, 3189.
